# Switching Response in Organic Electrochemical Transistors by Ionic Diffusion and Electronic Transport

**DOI:** 10.1002/advs.202404182

**Published:** 2024-07-25

**Authors:** Juan Bisquert, Baurzhan Ilyassov, Nir Tessler

**Affiliations:** ^1^ Instituto de Tecnología Química (Universitat Politècnica de València‐Agencia Estatal Consejo Superior de Investigaciones Científicas) Av. dels Tarongers València 46022 Spain; ^2^ Institute of Advanced Materials (INAM) Universitat Jaume I Castelló 12006 Spain; ^3^ Astana IT University Mangilik El 55/11, EXPO C1 Astana 010000 Kazakhstan; ^4^ Andrew & Erna Viterbi Department of Electrical and Computer Engineering Technion‐Israel Institute of Technology Haifa 32000 Israel

**Keywords:** organic transistor, hysteresis, transient, switching, ionic diffusion

## Abstract

The switching response in organic electrochemical transistors (OECT) is a basic effect in which a transient current occurs in response to a voltage perturbation. This phenomenon has an important impact on different aspects of the application of OECT, such as the equilibration times, the hysteresis dependence on scan rates, and the synaptic properties for neuromorphic applications. Here we establish a model that unites vertical ion diffusion and horizontal electronic transport for the analysis of the time‐dependent current response of OECTs. We use a combination of tools consisting of a physical analytical model; advanced 2D drift‐diffusion simulation; and the experimental measurement of a poly(3‐hexylthiophene) (P3HT) OECT. We show the reduction of the general model to simple time‐dependent equations for the average ionic/hole concentration inside the organic film, which produces a Bernards‐Malliaras conservation equation coupled with a diffusion equation. We provide a basic classification of the transient response to a voltage pulse, and the correspondent hysteresis effects of the transfer curves. The shape of transients is basically related to the main control phenomenon, either the vertical diffusion of ions during doping and dedoping, or the equilibration of electronic current along the channel length.

## Graphical abstract

An analytical model for the transient response of organic electrochemical transistors combines vertical ionic diffusion and horizontal electronic transport. A classification of transients and hysteresis effects is obtained, and the model is developed by experimental measurements and advanced simulations.

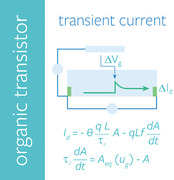

## 1 Introduction

The realization of the point contact transistor in 1947 marked the beginning of the transistor era and the development of various transistor structures aiming to replace the vacuum‐tube technology.^[^ ^1^ ^]^ The demonstration of the first microprocessor in 1971 by Intel turned the metal oxide semiconductor field effect transistor (MOSFET) into the dominant transistor technology. Interestingly, before founding Intel, Gordon Moore extrapolated 4 years of data points to predict that the number of transistors per chip would double every 2 years.^[^ ^2^ ^]^ This soon became the industry's target and what is known today as Moore's law. For many years, the law was followed by “simply” shrinking the size of the transistor. However, as the challenges accumulated, new transistor architectures were developed, and 3D integration became necessary. In parallel, a trend of improving performance not just by packing more transistors but also by performing the functions differently started evolving.^[^ ^3^ ^]^

The appearance of artificial intelligence and the notion of multi‐level logic has accelerated the development of multi‐level switches, the most famous of which is the memristor family.^[^ ^4^ ^]^ Recent developments show that the good‐old MOSFET transistor and its organic analog^[^ ^5^ ^]^ can also be turned into a multi‐level switch by embracing electrochemistry as part of the transistor's toolbox.^[^ ^6^, ^7^, ^8^ ^]^ Specifically, the electrochemical RAM (ECRAM) and the organic electrochemical transistor (OECT) use ionic and electronic conduction to establish new operation mechanisms, with the OECT also providing an interface to the biological world.^[^ ^9^ ^]^ Recently, three‐terminal devices have been investigated as programmable resistors for brain‐like computation. If the ion reservoir is left electrically open, the ions remain in the channel at the set conductivity, with a nonvolatile property. This device class includes ENODe (Electrochemical Neuromorphic Device), and EIS (Electrochemical Ionic Synapse) configurations.^[^ ^10^, ^11^, ^12^, ^13^, ^14^, ^15^ ^]^

It has been widely recognized that the dynamics of charge and ion transport exert a dominant influence on the operation of OECT.^[^ ^16^, ^17^, ^18^ ^]^ In particular slow ionic motion often creates a kinetic limiting effect.^[^ ^19^, ^20^ ^]^ These characteristic dynamics have an important influence on the switching time of the OECT, and on the memory effects for neuromorphic applications. An important feature that has been observed in OECTs is the hysteresis in the transfer curves.^[^ ^16^, ^21^, ^22^ ^]^

The analysis of the transient behavior of organic transistors has been developed by a standard distributed transmission line approach for both the injection of electronic currents from the drain and source and the injected ionic current from the electrolyte.^[^ ^23^, ^24^, ^25^, ^26^, ^27^, ^28^ ^]^ A particular realization is the Bernard and Malliaras model^[^ ^29^ ^]^ which has been widely used for the characterization of the transient response of the OECTs.^[^ ^24^, ^29^, ^30^, ^31^ ^]^ Recently we have developed a model of hysteresis in OECT^[^ ^32^ ^]^ that consists of an extension of the Bernards and Malliaras model by coupling the diffusion of intercalated ions explicitly. The analytical model provides some useful distinctions of hysteresis effects provided that the density of ions can be considered nearly homogeneous in the film. Here we establish a rigorous general formulation of the model and the suitable approximations that can be used to formulate the current response to different types of kinetic measurements, such as voltage scan at a constant rate, and sudden connection. Our method is to incorporate the diffusion of ions in the vertical direction in a transmission line formalism so that we can determine different kinetic times constants that govern the charging and discharging of the channel toward the steady‐state situation, and the correspondent hysteresis phenomena.

Below we apply the model investigation of the main hysteresis effects in both accumulation and depletion semiconductors.^[^ ^27^ ^]^ We also explore the realistic transient current and hysteresis effects using a 2D drift‐diffusion simulation model, that takes into account the ionic distribution and diffusion both in the organic film and the electrolyte.^[^ ^33^, ^34^, ^35^, ^36^, ^37^ ^]^ Furthermore we show some characteristic experimental results of a poly(3‐hexylthiophene) (P3HT) OECT, working in an accumulation mode. These results provide a consistent picture of the interaction of ionic and electronic effects that govern the major hysteresis effects observed in OECTs. The simulation allows one to explore different conditions of inhomogeneity, and a new mechanism is identified where the channel current follows the build‐up of the anion density at the solution/semiconductor interface.

## 2 Model

### 2.1 Geometry of the Model and Carrier Distribution

**Figure 1:**
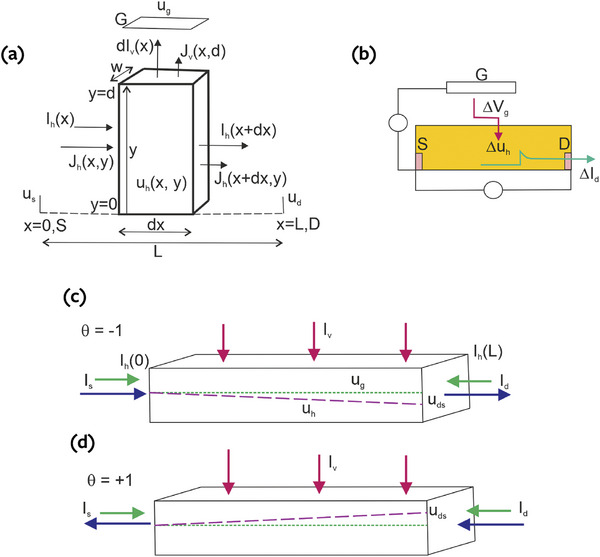
a) Scheme of the model for the transport inside the channel and ion exchange with the electrolyte. b) Scheme of the measurement of the transient response of the OECT. c,d) The blue current is the stationary electronic current $I_{d}^{dc}=I_{s}^{dc}$. The green arrows are the transient charging electronic currents. In a transient situation, the difference of green currents at D and S electrodes, *I_h_* (*L*) − *I_h_* (0), equals the total ion current entering the channel film (red currents). The voltage distribution in quasi‐equilibrium conditions is indicated for two situations: c) θ  =   − 1,  *u_ds_* < 0, and (d) θ  =   + 1,  *u_ds_* > 0.

The model shown in **Figure** **1**a considers a horizontal channel in direction *x* that spans from 0 to *x*  =  *L*, with horizontal current *I*_h_ and local voltage *u*_h_. The vertical direction in the channel is *y*. Note that current exiting the drain at *x*  =  *L* is considered positive.

If *z* is the effective cation density, then by local electroneutrality, the local hole density is

(1) $$p=p_{0}-z$$

where *p*_0_ in an intrinsic density due to doping. The density *z* depends on the concentrations of intercalated cations *m* and anions *a*

(2) z=m−a

In a volume element of length *dx* the interface with the electrolyte is at *y*  =  *d*. We define an average concentration per unit horizontal distance by the vertical integration of Equations (1) and (2)

(3) $$Z=w\int_{0}^{d}z\;dy=w\int_{0}^{d}m\;dy-w\int_{0}^{d}a\;dy$$

Hence

(4) Z=M−A

and the hole density is

(5) $$P=w\int_{0}^{d}p\;dy=P_{0}-Z$$

An equilibrium concentration of ions

(6) $$Z_{\mathrm{eq}}=Z\left(u_{g}\right)$$

is obtained when the film is charged homogeneously at the gate potential *u_g_*. This function can be obtained by electrochemical methods for a given type of film^[^ ^38^, ^39^, ^40^, ^41^, ^42^ ^]^ as commented in Section 2.4.

Two different OECTs operation modes are called depletion and accumulation.^[^ ^27^ ^]^ In accumulation, the device is normally OFF in the absence of a gate bias (i.e., the semiconducting polymer is initially in its neutral stage). In depletion, the active material in the channel is initially doped and the OECT is naturally in its ON state and is turned OFF upon application of a gate voltage (dedoping of the channel). Below we consider two types of situations:

- In accumulation (undoped semiconductor) *P*_0_ =  0, *Z*  =   − *A*, *P*  =  *A*.
- In depletion (doped semiconductor) *A*  =  0, *Z*  =  *M*, *P*  = *P*_0_  − *M*.

### 2.2 Definition of Currents

The target measurement we wish to describe is the transient drain current with respect to a step of gate voltage indicated in Figure 1b. We consider the properties of currents in the channel film.

The horizontal flux of hole carriers is given by the drift transport in the electrical field

(7) $$J_{h}\;\left(x,y\right)=-p\left(x,y\right)\mu_{p}\frac{du_{h}}{dx}$$

Here µ_p_ is the mobility. More general transport conditions have been investigated,^[^ ^43^ ^]^ but here we use the standard approach. The horizontal current is

(8) $$I_{h}\;\left(x\right)=q\;w\int_{0}^{d}J_{h}\left(x,y\right)\;dy$$

where *q* is the elementary charge. Therefore

(9) $$I_{h}\;\left(x\right)=-qP\left(x\right)\mu_{p}\frac{du_{h}}{dx}$$

In Equation (9) the model is restricted to situations in which the vertical gradient of *u*_h_ is not large.

The current across the top interface of the volume element is exclusively ionic. An outward flux of positive ions $J_{v}^{m}$ and negative ions $J_{v}^{a}$ produces a vertical current *dI_v_* due to the area *w* *dx*, of value

(10) $$dI_{v}\;\left(x\right)=q\;w\left(J_{v}^{m}\left(x,d\right)-J_{v}^{a}\left(x,d\right)\right)\;dx$$

The current conservation in the volume element of Figure 1a is

(11) $$I_{h}\left(x+dx\right)-I_{h}\left(x\right)+dI_{v}\;\left(x\right)=0$$

Therefore

(12) $$\frac{\partial I_{h}}{\partial x}dx=-qw\left[J_{v}^{m}\left(x,d\right)-J_{v}^{a}\left(x,d\right)\right]dx$$

The simple application of Kirchhoff's rules produces the two classical equations of a transmission line model, (10) and (12).^[^ ^44^ ^]^ This approach has been developed in field‐effect transistors^[^ ^23^ ^]^ and in OECT.^[^ ^24^, ^25^, ^26^, ^27^ ^]^

The model requires a completion by stating the vertical flux, *J*_v_. The flux depends on the voltage in the electrolyte just outside the interface, that is the gate voltage *u_g_*, and on the voltage inside the film, *u*_h_. This is developed in Section 2.5.

### 2.3 Capacitive Coupling

In the previous theories of OECTs the ion concentration inside the film *z* is coupled capacitively to the voltage difference across the interface. The total ionic charge is *q* *w* *d* *dx* *z*. Considering the double‐layer capacitance per unit area in the top surface, *c_d_*, we have^[^ ^26^, ^29^, ^31^ ^]^

(13) $$q\;d\;z\left(x\right)\;w\;dx=c_{d}\;\left(u_{g}-u_{h}\left(x\right)\right)\;w\;dx$$

In steady state, the horizontal current is constant, *I_h_* (*x*)  = *I_ds_*. The drain‐source voltage is

(14) $$u_{ds}=u_{h}\;\left(L\right)-u_{h}\left(0\right)$$

and the integration of Equation (9) gives

(15) $$I_{ds}=-\frac{q\;d\;w\;\mu }{L}\left[p_{0}-\frac{c_{d}}{qd}\left(u_{g}-\frac{1}{2}u_{ds}\right)\right]u_{ds}$$

This is a standard result.^[^ ^29^, ^30^ ^]^ Later, it was discovered^[^ ^19^, ^30^, ^45^ ^]^ that in OECT the capacitance *c_d_* shows a correlation with length. Then the volume capacitance

(16) $$c^{*}=\frac{c_{d}}{d}$$

is the constant quantity, since the ions fill the whole element in Figure 1a. This observation leads one to consider the related ideas of ion intercalation in battery and electrochromic materials^[^ ^46^, ^47^, ^48^ ^]^ and in conducting polymers,^[^ ^39^, ^40^ ^]^ that have been broadly investigated. But then, a different formalism than Equation (13) is required to account for the intercalation of ions from the electrolyte, based on the ion diffusion concept.^[^ ^49^ ^]^ Diffusion itself generates a chemical capacitance due to the intercalation process,^[^ ^50^, ^51^, ^52^, ^53^ ^]^ so that the capacitive effect arises naturally.

### 2.4 The Chemical Capacitance and the Thermodynamic Equilibrium Functions

As a preliminary step to the generalization of the channel dynamics including ionic diffusion, it is important to remark on the idea of the chemical capacitance, that appears in the diffusion transport.^[^ ^54^ ^]^ The chemical capacitance of a species^[^ ^50^, ^51^, ^53^ ^]^ is obtained by the derivative of the thermodynamical function of the density with respect to the electrochemical potential. For the ions in the organic film

(17) $$c_{\mu }=q\frac{dZ_{eq}}{du_{g}}$$

This equation expresses the volume capacitance c* mentioned in (16) in the more general denomination of the chemical capacitance *c*_µ_ that is measured in the electrochemistry of organic conductors.^[^ ^55^, ^56^, ^57^ ^]^ The bulk origin of the ionic capacitance is well established in organic films.^[^ ^58^ ^]^ Furthermore, it is obtained a relation of the chemical capacitance and the density of states (DOS) *g*,^[^ ^56^, ^57^ ^]^ as follows

(18) $$c_{\mu }=q\;g\left(qu_{g}\right)$$

Therefore, the chemical capacitance gives a direct measure of the DOS. It also provides a figure of merit of OECT.^[^ ^45^ ^]^

For an anion density that decreases at increasing *u*_g_ the chemical capacitance is

(19) $$c_{\mu }=-q\frac{dA_{eq}}{du_{g}}$$

The sign in Equation (19) is due to the fact that when the potential *u_g_* becomes more negative, the concentration of anions increases, so that *c*_µ_ is positive.

Many forms of *Z_eq_* (*u_g_*) are possible according to the properties of the organic film.^[^ ^41^, ^42^, ^46^, ^47^, ^55^, ^59^, ^60^ ^]^ In coherence with the drift‐diffusion simulations described later, here we use the density of states (DOS) for inserted anions

(20) $$g_{a}\;\left(qu_{g}\right)=-\frac{dA_{eq}}{d\left(qu_{g}\right)}=\frac{A_{0}}{k_{B}T}{\left[\frac{q\left(u_{v}-u_{g}\right)}{k_{B}T}\right]}^{1/2}$$

The *k*_B_ is the Boltzmann's constant, *T* the absolute temperature, *u*_v_ the valence edge potential, *A*_0_ a density per length, from the volume density *a*_0_ = *A*_0_ /*wd*. This is the DOS used for holes in the simulations below, which by electroneutrality applies to ions as well. Accordingly, the thermodynamic function is

(21) $$A_{eq}\;\left(u_{g}\right)=-\int_{u_{g}}^{u_{v}}g_{a}\left(x\right)dx=\frac{2}{3}\;A_{0}{\left[\frac{q\left(u_{v}-u_{g}\right)}{k_{B}T}\right]}^{3/2}$$

For insertion of cations

(22) $$g_{m}\;\left(qu_{g}\right)=-\frac{dM_{eq}}{d\left(qu_{g}\right)}=\frac{M_{0}}{k_{B}T}{\left[\frac{q\left(u_{g}-u_{c}\right)}{k_{B}T}\right]}^{1/2}$$

*M*_0_ is a density per length, from the volume density *m*_0_ = *M*_0_ /*wd*. For a cation density that increases at increasing *u*_g_ the chemical capacitance

(23) $$c_{\mu }=q\frac{dM_{eq}}{du_{g}}$$

is positive.

The thermodynamic function is

(24) $$M_{eq}\;\left(u_{g}\right)=-\int_{u_{c}}^{u_{g}}g_{m}\left(x\right)dx=\frac{2}{3}\;M_{0}{\left[\frac{q\left(u_{g}-u_{c}\right)}{k_{B}T}\right]}^{3/2}$$

The general conclusions on time transient and hysteresis derived below are not dependent on the specific expressions *A_eq_*, *M_eq_*, provided that these functions are such that the chemical capacitances remain positive.

### 2.5 Diffusion of Intercalated Ions

Here we aim to introduce the diffusion dynamics that have applications for solid‐state or electrochemical ionic diffusion in organic and inorganic materials.^[^ ^11^, ^12^ ^]^ This analysis departs from the previous treatments in the literature based on Equation (13),

The diffusion of ions in the film is described by the conservation equation

(25) $$\frac{\partial z}{\partial t}=-\frac{\partial J_{v}}{\partial y}$$

and the Fick's law that states that the ion flux is proportional to the ion diffusion coefficient *D_ion_* and the gradient of the concentration

(26) $$J_{v}=-D_{ion}\frac{\partial z}{\partial y}$$

Considering that the bottom layer in Figure 1a is blocking ions, *J_v_* (*y*  =  0) =  0, the integral of (25) gives

(27) $$w\int_{0}^{d}\frac{\partial z}{\partial t}dy=-wJ_{v}\left(x,d\right)$$

(28) $$\frac{\partial Z}{\partial t}=-wJ_{v}\left(x\right)$$

This last equation incorporates the assumption that the ionic charge vertically injected is compensated by the electronic charge of the opposite sign from the immediate surroundings. This is the universal assumption used in intercalation systems such as Li‐ion batteries, that are described by the ion diffusion rate at the boundary with the electrolyte.^[^ ^61^ ^]^ However, this assumption requires that the dielectric relaxation time is short, which occurs when sufficient electronic conductivity is available. In more detailed treatments one should consider the coupling of ionic and electronic local currents^[^ ^62^, ^63^ ^]^ as further discussed in Section 3.3 and 6.3.

The conservation equation for cations and anions gives the results

(29) $$wJ_{v}^{m}\left(x\right)=-\frac{\partial M}{\partial t}$$

(30) $$wJ_{v}^{a}\left(x\right)=-\frac{\partial A}{\partial t}$$

From Equations (9) and (12) the equations of the transmission line model can be stated

(31) $$I_{h}\;\left(x\right)=-q\left(P_{0}-M+A\right)\mu_{p}\frac{du_{h}}{dx}$$

(32) $$\frac{\partial I_{h}}{\partial x}=q\left(\frac{\partial M}{\partial t}-\frac{\partial A}{\partial t}\right)$$

### 2.6 Current and Electronic Transit Time

For a small *u_ds_* that produces a uniform electrical field, the current (30) can be written

(33) $$I_{h}\;\left(x\right)=-q\mu_{p}\frac{u_{ds}}{L}\left(P_{0}-M\left(x\right)+A\left(x\right)\right)$$

By integration of Equation (32) we obtain

(34) $$I_{h}\left(L\right)-I_{h}\left(0\right)=q\int_{0}^{L}\left(\frac{\partial M}{\partial t}-\frac{\partial A}{\partial t}\right)dx$$

We define drain and source current as

(35) $$I_{s}=I_{h}\;\left(0\right)$$

(36) $$I_{d}=I_{h}\;\left(L\right)$$

Drain and source currents are not equal in the transient condition, due to the charging by the vertical current of intercalation, as shown in Figure 1c,d.

To confirm the interpretation of signs of Equation (34) consider an electronically blocking source contact, *I*_h_ (0) =  0. We inject a pulse of anions and Equation (34) gives

(37) $$I_{h}\;\left(L\right)=-q\int_{0}^{L}\left(\frac{\partial A}{\partial t}\right)dx\;\left(\mathrm{blocking}\;\mathrm{source}\right)$$

If we increase the concentration of anions, by more negative *u*_g_, then ∂*A*/∂*t* > 0, hence the current (37) is negative, as holes enter the film from the right side in Figure 1c.

Let us define the sign of the voltage drop as

(38) $$\theta =\frac{u_{ds}}{\left|u_{ds}\right|}\;$$

The two cases θ ± 1 that we describe in the following are indicated in Figure 1c,d. The standard operation of a p‐type transistor corresponds to *u_ds_* < 0 or θ  =   − 1, as the hole carriers are injected from the source to the channel (*I_d_* is positive). Studying both signs of θ, within the scope of this model, is equivalent to studying both the drain (θ  =   − 1) and source (θ  =   + 1) currents, which is most important for transient studies, as they can be separately measured and have distinct behaviors.^[^ ^23^, ^64^ ^]^

We introduce the electronic transit time along the channel length^[^ ^29^ ^]^

(39) $$\tau_{e}=\frac{L^{2}}{\mu_{p}\left|u_{ds}\right|}$$

The current (33) is expressed

(40) $$I_{h}\;\left(x\right)=-\theta \frac{qL}{\tau_{e}}\left(P_{0}-M\left(x\right)+A\left(x\right)\right)$$

### 2.7 A Simple Solution: Bernard–Malliaras Model for the Undoped Situation

Assume an undoped sample in which the hole density is due to intercalated anions *A*. To obtain a functional model for the transient currents, a solution of Equations (32) and (40) must be obtained. We can combine these equations as

(41) $$\frac{\partial A}{\partial x}=\theta \frac{\tau_{e}}{L}\frac{\partial A}{\partial t}$$

and we obtain the integral

(42) $$A\left(L\right)-A\;\left(0\right)=\theta \frac{\tau_{e}}{L}\int_{0}^{L}\frac{\partial A}{\partial t}dx$$

We allow for holes to be injected from both contacts (Figure 1c). To obtain the source and drain currents by Equations (35), (36) and (40), the separate *A*(*L*),  *A*(0) are needed.

Following Bernard and Malliaras^[^ ^29^ ^]^ we split the left‐hand side of (42) into two different parts, by the fractional constant 0 < *f* < 1

(43) $$A\;\left(L\right)=A_{av}+\theta f\frac{\tau_{e}}{L}\int_{0}^{L}\frac{\partial A}{\partial t}dx=A_{av}+\theta f\tau_{e}\frac{\partial A_{av}}{\partial t}$$

(44) $$A\;\left(0\right)=A_{\mathrm{av}}+\theta \left(1-f\right)\frac{\tau_{e}}{L}\int_{0}^{L}\frac{\partial A}{\partial t}dx=A_{av}+\theta \left(1-f\right)\tau_{e}\frac{\partial A_{av}}{\partial t}$$

The *A*_av_ is an average concentration taken by a suitable procedure. Different integration schemes^[^ ^24^, ^65^, ^66^ ^]^ to justify the value of *f* are summarized in ref. [19] Removing the subscript *av*, the drain current in (36) is

(45) $$I_{h}\;\left(L\right)=-\theta \frac{qL}{\tau_{e}}A-qfL\frac{dA}{dt}$$

We note that the Bernards‐Malliaras Equation (45) is a statement of the current conservation in the film, by dividing Equation (34) into two separate parts.

### 2.8 Doped Semiconductor

We take the case *A*  =  0, *Z*  =  *M*, *P*  = *P*_0_  − *M*. By similar calculations as before

(46) $$I_{h}=-\theta \frac{q\;L}{\tau_{e}}\left(P_{0}-M\right)$$

(47) $$\frac{\partial M}{\partial x}=\theta \frac{\tau_{e}}{L}\frac{\partial M}{\partial t}$$

(48) $$I_{h}\;\left(L\right)=-\theta \frac{qL}{\tau_{e}}\left(P_{0}-M\right)+qfL\frac{dM}{dt}$$

If a pulse ∂*M*/∂*t* > 0 is injected a positive hole current *I_d_* =  *qfL*∂*M*/∂*t* > 0 has to leave the channel.

### 2.9 Local Diffusion Effect

In addition to the current conservation, it must be stated how the *A* evolves with time, which is not given by Equation (45). The previous models proposed in the literature, based on Equation (45), have not considered explicitly the ion diffusion process. For a rigorous solution, one can solve Equation (12) coupled to Equation (25). This is a complex problem that will generate another (vertical) transmission line at each point *x*.^[^ ^67^ ^]^

To obtain a physically meaningful but approximated solution, we have suggested^[^ ^32^ ^]^ that the boundary flux is given by a gradient of concentration associated with different electrochemical potentials of the volume element of Figure 1a, from the bottom *u_h_* to the top *u_g_*. Then

(49) $$J_{v}^{a}\;\left(x,d\right)=-D_{ion}\frac{\partial a}{\partial y}\approx \frac{D_{X}}{d}\left[a\left(u_{h}\right)-a\left(u_{g}\right)\right]$$

Therefore

(50) $$\frac{\partial A}{\partial t}=\frac{D_{ion}}{d^{2}}\;\left[A\left(u_{g}\right)-A\left(u_{h}\right)\right]$$

We define the transit time for diffusion

(51) $$\tau_{d}=\frac{d^{2}}{D_{ion}}$$

Using the notion that the chemical capacitance is charged by a diffusion current, we can express Equation (29) as

(52) $$\frac{\partial A}{\partial t}=\frac{1}{\tau_{d}}\;\left(A_{eq}\left(u_{g}\right)-A\left(u_{h}\right)\right)$$

Equations (45) and (52) are the model introduced in ref. [32] that here has been rigorously justified.

Similarly for the insertion of cations

(53) $$\frac{\partial M}{\partial t}=\frac{1}{\tau_{d}}\;\left(M_{eq}\left(u_{g}\right)-M\left(u_{h}\right)\right)$$

Note that the diffusion of ions in Equation (49) requires a flow of compensating holes. If the time associated with hole compensation is τ_*v*_ then it is required that τ_*v*_ ≪ τ_*d*_, as commented earlier, to satisfy the electroneutrality condition. If only a vertical flow is needed for the charge compensation then

(54) $$\tau_{v}=\frac{d^{2}}{\mu_{p}\left(u_{g}-u_{h}\right)}$$

Based on the general transmission line approach of Equations (9) and (12) one can add different effects that influence the transient behavior, for example, a distributed interfacial capacitance as Equation (13)^[^ ^24^, ^68^ ^]^ and a series resistance in the electrolyte.^[^ ^25^ ^]^ These improvements are commented on in Section 3.3. The realistic 2D flows of charge will be discussed in the simulations in Section 6.

## 3 Transient Switching Dynamics

We start the study of transient behavior based on the model of Equations ((45), (52)).^[^ ^32^ ^]^ Let us summarize the structure of the model. We consider an accumulation semiconductor, *P*_0_ =  *M*  =  0, for a *u_ds_* small voltage, in which *A*(*x*, *t*) is nearly homogeneous, with equilibrium function *A_eq_* given in Equation (20). In the stationary dc current the cation density obtains the value of equilibrium according to the gate voltage *A_eq_* (*u_g_*). As mentioned before the voltage applied to the gate is *u_g_*. The voltage in the channel is *u_h_*. The model for any time‐dependent situation consists on two equations for the three variables *I_d_*, *u_g_*, *A*.

(55) $$I_{d}=-\theta \frac{q\;L}{\tau_{e}}A-qLf\frac{dA}{dt}$$

(56) $$\tau_{d}\;\frac{dA}{dt}=A_{eq}\;\left(u_{g}\right)-A$$

Here the concentration *A* can be regarded as *A*(*u_h_*), a unique function of the inner potential.

### 3.1 The Linear Equations for a Small Perturbation

The properties of switching will be investigated by a small step of the external voltage Δ*V* at *t*  =  0 that changes the gate voltage from an initial value *u*_*g*0_

(57) $$u_{g}=u_{g0}+\Delta V$$

The internal voltage in the film changes as

(58) $$u_{h}\;\left(t\right)=u_{g0}+\Delta v\left(t\right)$$

where Δ*v* (*t*  =  0) =  0 and Δ*v* (*t*  =  ∞) =  Δ*V*. Considering the chemical capacitance of Equation (18), the change of the ion densities in Equations (55) and (56) is

(59) $$A\;\left(u_{g0}+\Delta v,\;t\right)=A_{eq}\;\left(u_{g0}\right)-\frac{c_{\mu }}{q}\Delta v\;\left(t\right)$$

(60) $$A_{eq}\;\left(u_{g0}+\Delta V\right)=A_{eq}\;\left(u_{g0}\right)-\frac{c_{\mu }}{q}\Delta V$$

Thus Equation (56) takes the form

(61) $$\tau_{d}\;\frac{d\Delta v}{dt}=\Delta V-\Delta v$$

The total step of the current is

(62) $$I_{fin}=I_{in}+\theta \frac{c_{\mu }L}{\tau_{e}}\Delta V$$

(63) $$I_{in}=-\theta \frac{q\;L}{\tau_{e}}A_{eq}\left(u_{g0}\right)$$

with *I_fin_* and *I_in_* being the final and initial currents. The total quantity of electronic charge

(64) $$\Delta Q=\theta Lc_{\mu }\Delta V$$

enters the channel for the new equilibrium situation.

Now the time‐dependent current of Equation (55) can be expressed

(65) $$I_{d}=-\theta \frac{qL}{\tau_{e}}\;A_{eq}\left(u_{g0}\right)+\theta Lc_{\mu }\frac{1}{\tau_{e}}\Delta v+Lc_{\mu }f\frac{d\left(\Delta v\right)}{dt}$$

And using Equation (61) we have

(66) $$I_{d}=-\theta \frac{qL}{\tau_{e}}\;A_{eq}\left(u_{g0}\right)+\theta Lc_{\mu }\frac{1}{\tau_{e}}\Delta v+Lc_{\mu }f\frac{1}{\tau_{d}}\left(\Delta V-\Delta v\right)$$

The transient current is composed of two parts. The second term corresponds to the horizontal transport of the injected charge. The third term is the compensation of the vertical injection of the charge. Both terms, when combined, inject the total charge (64).

Let us write the Equation (66) in the form

(67) $$I_{d}=-\theta \frac{qL}{\tau_{e}}\;A_{eq}\left(u_{g0}\right)+Lc_{\mu }f\frac{1}{\tau_{d}}\Delta V+Lc_{\mu }\left(\frac{\theta }{\tau_{e}}-\frac{f}{\tau_{d}}\right)\Delta v$$

We remark that the current step at the first instant is

(68) $$\Delta I_{d}\left(t=0\right)=+Lc_{\mu }f\frac{1}{\tau_{d}}\Delta V$$

We can analyze these effects by using the explicit dependence on time. Equation (61) gives

(69) $$\Delta v=\Delta V\left(1-e^{-t/\tau_{d}}\;\right)$$

Therefore

(70) $$\begin{array}{ccc} I_{d}\left(t\right) & = & -\theta \frac{qL}{\tau_{e}}\;A_{eq}\left(u_{g0}\right)+Lc_{\mu }f\frac{1}{\tau_{d}}\Delta V\\ & & +\;Lc_{\mu }\left(\frac{\theta }{\tau_{e}}-\frac{f}{\tau_{d}}\right)\Delta V\left(1-e^{-t/\tau_{d}}\;\right) \end{array}$$

**Figure 2:**
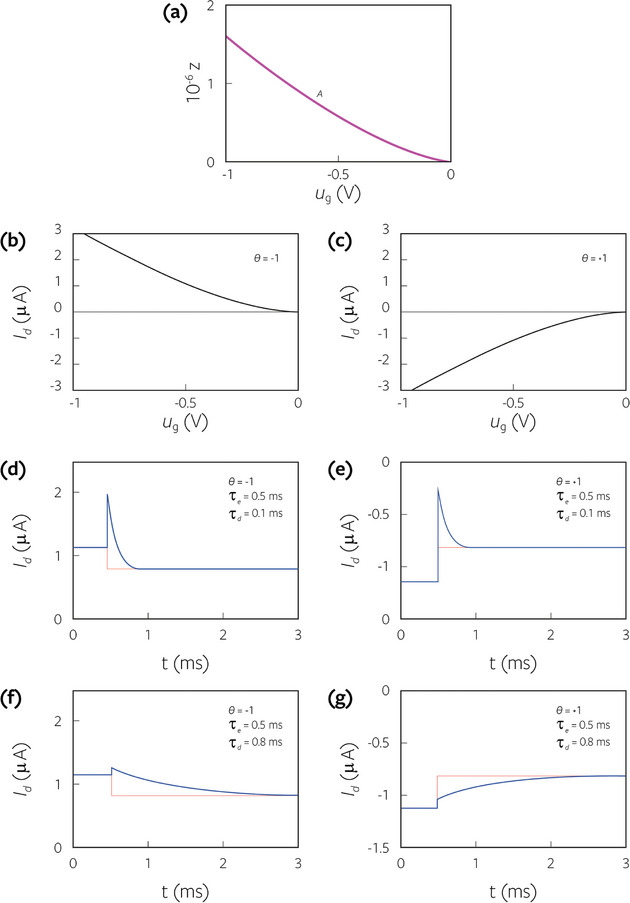
Undoped semiconductor. a) Equilibrium anion density in the film. The stationary current b,c) according to the sign θ  = *u_ds_* /|*u_ds_* | and (d‐g) the possible four different types of transient response with respect to time, for a step voltage at *V*_0_ =   −0.5 *V* and Δ*V*  =  0.1 *V* at *t*_0_ =  0.5 ms. The brown line is an immediate current response. We take the parameters of Table 1, τ_*e*_ =  0.5 ms, *a*_0_ =  6.2 × 10^18^ cm^−3^, *A*_0_ =  6.2 × 10^12^ m^−1^, *u_v_* =  0, *f*  =  0.5 and τ_*d*_ as indicated.

**Figure 3:**
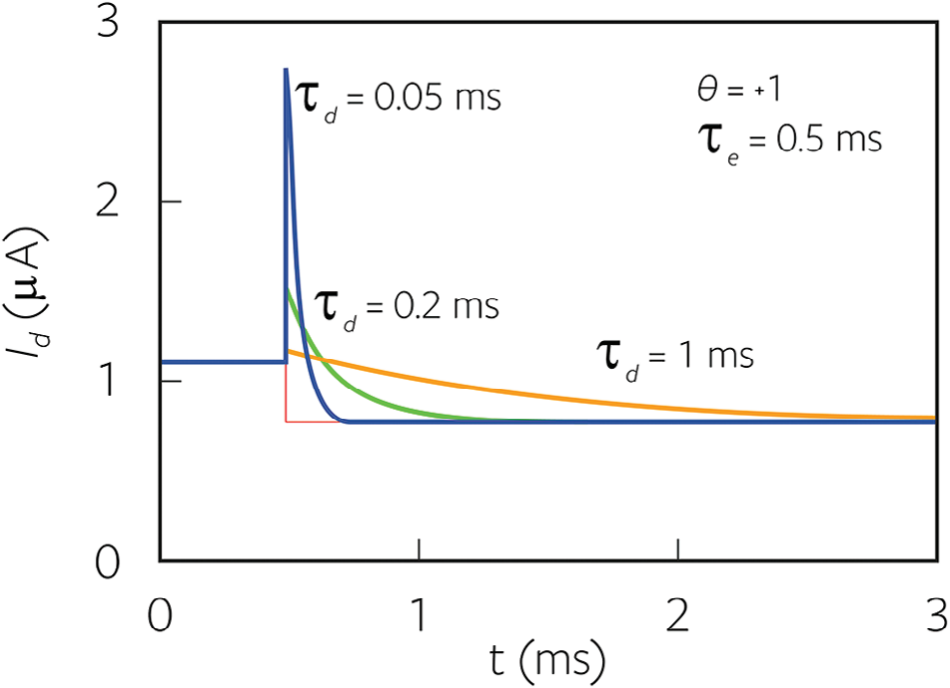
Switching transients for an undoped semiconductor, same as in Figure 2 for several values of τ_*d*_.

In **Figure** **2** we show the transient response for the parameters outlined in **Table** **1**. There are four different cases, distinguished by τ_*e*_ < τ_*d*_ or vice versa, and also by the sign of the current θ, that makes the step either increase or decrease the current in the final state. The initial value of the transient current is only dependent on Δ*V*/τ_*d*_, by Equation (68). The characteristic time for the transient is the vertical ion diffusion time τ_*d*_. This is shown in several cases in **Figure** **3**. The final value of the current only depends on τ_*e*_, Equation (62). The area of the curve corresponds to (64), so that in Figure 3 the spike is higher for shorter charging time τ_*d*_. If τ_*e*_ <  τ_*d*_, Figure 2g, the initial spike is smaller than the equilibrium current, hence the current does not make a spike but increases since the first instant.^[^ ^29^ ^]^ In the transition between Figures 2e and 2g, the transient almost vanishes, as remarked in ref. [24].

**Table 1:** Parameters used in the simulations.

Channel length	*L*	50 µm
thickness	*d*	100 nm
width	*w*	10 µm
Hole mobility	µ_*p*_	0.02 cm^2^ Vs^−1^
Source‐drain voltage	|*u_ds_* |	0.1 V
Thermal energy	*k_B_T*	0.026 V

### 3.2 Doped Semiconductor

Here *A*  =  0, *Z*  =  *M*, *P*  = *P*_0_  − *M*. The equations for the general transient behavior are

(71) $$I_{h}\;\left(L\right)=-\theta \frac{qL}{\tau_{e}}\left(P_{0}-M\right)+qfL\frac{dM}{dt}$$

(72) $$\tau_{d}\;\frac{dM}{dt}=M_{eq}\;\left(u_{g}\right)-M$$

For a small voltage step, Δ*V* the internal voltage in the film changes as

(73) $$u_{h}\;\left(t\right)=u_{g0}+\Delta v\left(t\right)$$

The total step of the current is

(74) $$I_{fin}=I_{in}+\theta \frac{c_{\mu }L}{\tau_{e}}\Delta V$$

Here *I_in_* is the initial current,

(75) $$I_{in}=-\theta \frac{qL}{\tau_{e}}\left(P_{0}-M_{eq}\right)$$

The time‐dependent current is

(76) $$I_{d}=-\theta \frac{qL}{\tau_{e}}\;\left(P_{0}-M\left(u_{g0}\right)\right)+\theta Lc_{\mu }\frac{1}{\tau_{e}}\Delta v+fLc_{\mu }\frac{d\left(\Delta v\right)}{dt}$$

This can be written

(77) $$\begin{array}{c} I_{d}=-\theta \frac{qL}{\tau_{e}}\;\left(P_{0}-M\left(u_{g0}\right)\right)+fLc_{\mu }\frac{1}{\tau_{d}}\Delta V+Lc_{\mu }\left(\frac{\theta }{\tau_{e}}-\frac{f}{\tau_{d}}\right)\Delta v \end{array}$$

Therefore

(78) $$\begin{array}{ccc} I_{d}\;\left(t\right) & = & -\theta \frac{qL}{\tau_{e}}\;\left(P_{0}-M\left(u_{g0}\right)\right)+fLc_{\mu }\frac{1}{\tau_{d}}\Delta V\\ & & +\;Lc_{\mu }\left(\frac{\theta }{\tau_{e}}-\frac{f}{\tau_{d}}\right)\Delta V\left(1-e^{-t/\tau_{d}}\;\right) \end{array}$$

The initial step is again given by Equation (68)

**Figure 4:**
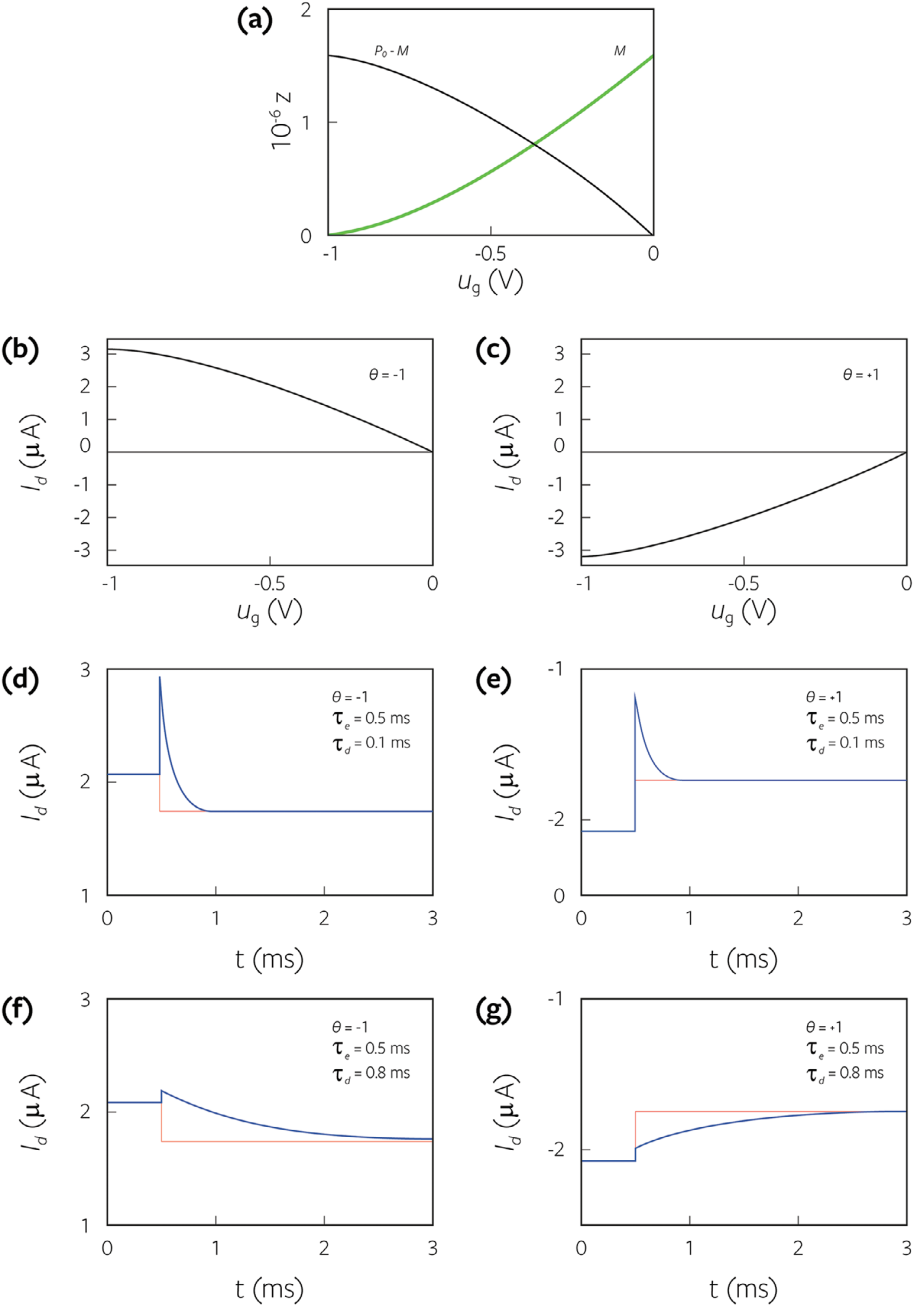
Doped semiconductor. a) Ionic density. b,c) The stationary current according to the sign θ  = *u_ds_* /|*u_ds_* | and d–g) the possible four different types of transient response with respect to time, for a step voltage at *V*_0_ =   − 0.5 *V* and Δ*V*  =  0.1 *V*. The brown line is an immediate current response. We take the parameters of Table 1, *u_c_* =   −1 V, τ_*e*_ =  0.5 ms, *m*_0_ =  6.2 × 10^18^ cm^−3^, *M*_0_ =  6.2 × 10^12^ m^−1^, *P*_0_ =  160 *M*_0_, *f*  =  0.5 and τ_*d*_ as indicated.

The four possible types of decays are shown in **Figure** **4**. For a better comparison of doped and undoped cases, we set the DOS parameter *u_c_* =   −1 V so that *I_d_* =  0 at the origin. The depletion case is obtained by shifting the curves to the right by *u_c_* =  0 V.

### 3.3 Interpretation of Transients

**Figure 5:**
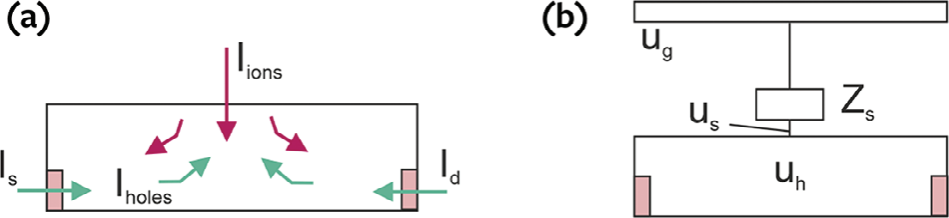
a) Red and green arrows represent the transient currents of ions and holes, respectively. In the transient charging, there are diagonal currents of ions and holes, to establish the electroneutrality. These currents can be controlled by either diffusion or drift, and the resistance will be larger for the species with the lowest local conductivity. b) Effect of electrical impedances at the electrolyte and its interfaces, combined into a series element of impedance *Z_s_*.

Figure 1b summarizes the model that we have developed. The measurement consists of applying a voltage pulse to the gate and recording the current extracted at the drain contact, while both gate voltage and drain current are measured with respect to the source electrode.^[^ ^66^, ^69^ ^]^ Of course, the physical situation can be complicated in several respects, compared to the simplified analytical model, which has to be clarified experimentally. This is outlined in **Figure** **5**a. The entrance of ions instigated by the change of gate voltage induces the uptake of holes from S and D electrodes. A complex pattern of currents is formed, in which

1. The current of each carrier can be dominated by either drift or diffusion.
2. The carriers are coupled by local electroneutrality, with the charge difference resulting in electrical fields according to the Poisson equation.
3. Which species is moving predominantly, is determined by the local conductivities. It can be either electrons or holes, causing diagonal currents as in Figure 5a.

These questions have been amply studied in the electrochemistry of conducting polymers,^[^ ^70^ ^]^ and applying multiple carrier transmission lines.^[^ ^70^, ^71^ ^]^ In the OECTs there could be a limiting horizontal transport of ions, or vertical transport of holes, as mentioned in Equation (54).^[^ ^18^, ^43^, ^72^, ^73^ ^]^ The implications of breaking quasineutrality are discussed in Section 6.3.

Despite the complexity of these transport processes, we have provided a solution to the problem of the transient as outlined in Figure 1c,d. This approach is based on the combination of Bernards‐Malliaras simplification of the transient conservation equation, and the analysis of ion diffusion. The model thus considers explicitly the interior of the channel. We summarize the properties of the model in the following, and in Section 6 the more general realistic transport will be considered, based on drift‐diffusion simulations.

First, we adopt a separation of two orthogonal currents, according to the principal nature of the electrodes: D and S are selective to electrons, while the top surface of the channel is selective to ions. Therefore, the vertical current is an ionic current assuming that the vertical electronic currents can be neglected. While the horizontal current along the channel is electronic.

When the Δ*V_g_* pulse is applied, it induces a variation of the internal voltage *u_h_*, as indicated in Equation (61). Consequently, we obtain a solution *u_h_* (*t*) that kinetically depends only on the vertical current, which in the model is defined by the ion diffusion effect. This is given in Equation (69). The transient duration depends only on τ_*d*_. This condition needs to be extended if there are additional elements in the vertical pathway, as in Figure 5b. We show a series element of impedance *Z_s_* that will have a capacitive and resistive component, e.g., a distributed interfacial capacitance as Equation (13),^[^ ^24^, ^68^ ^]^ and a series or charge transfer resistance.^[^ ^25^ ^]^ The^[^ ^24^ ^]^ surface voltage is *u_s_* and the current in the electrolyte is determined by the overall impedance *Z_s_* and the potential difference (*u_g_* − *u_s_*). These external elements can ultimately influence the relaxation time. This issue requires a detailed study that will be presented elsewhere. We have already remarked that a special solution exists in which the last term of Equation (78) vanishes,^[^ ^24^ ^]^ which is an exception to the transient being generally controlled by the diffusion characteristic time τ_d_.

In response to Δ*V_g_* pulse, the *I_d_* current undergoes a variation, as it is fully modulated by *u_h_*, Equation (55). We thus obtain the solution in Equation (70), where the exponential decay depends on the ion diffusion characteristic time, while the strength and sign of the decay are modulated by both ionic and electronic characteristics.

In summary, the classification of transients in Figures 2 and 4 is associated with the delay caused by the ion diffusion. In practice, this model can be generalized to account for additional electrical limitations, as shown in Figure 5b.

## 4 Hysteresis Effects

### 4.1 Undoped Semiconductor

**Figure 6:**
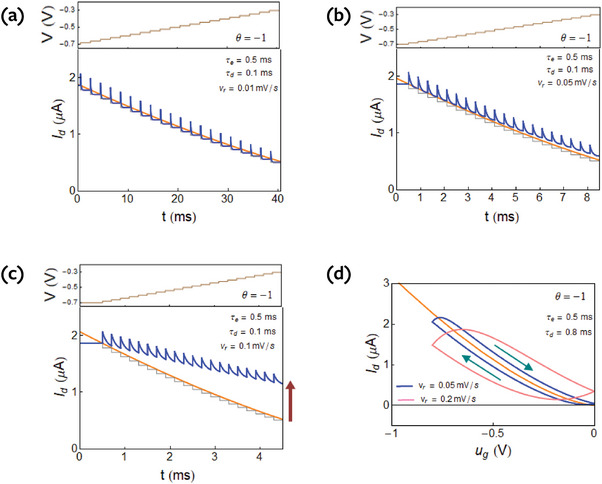
Undoped semiconductor for θ  =   −1 (u_ds_ < 0). a–c) Increasing the drain current by small voltage steps Δ*V*  =  0.02 *V* starting at *V*_0_ =   −0.7 V (*t*_0_ =  0.5 s) with velocity *v_r_* at time steps Δ*t*  =  Δ*V*/*v_r_*, at increasingly faster rates from (a) to (c). The grey step line is an immediate current response, the orange line is the equilibrium current‐voltage curve, and the red arrow in (c) is the final hysteresis current. d) Continuous hysteresis loops at the indicated scan rates. The same parameters as Figure 2 and τ_*d*_, τ_*e*_ as indicated.

**Figure 7:**
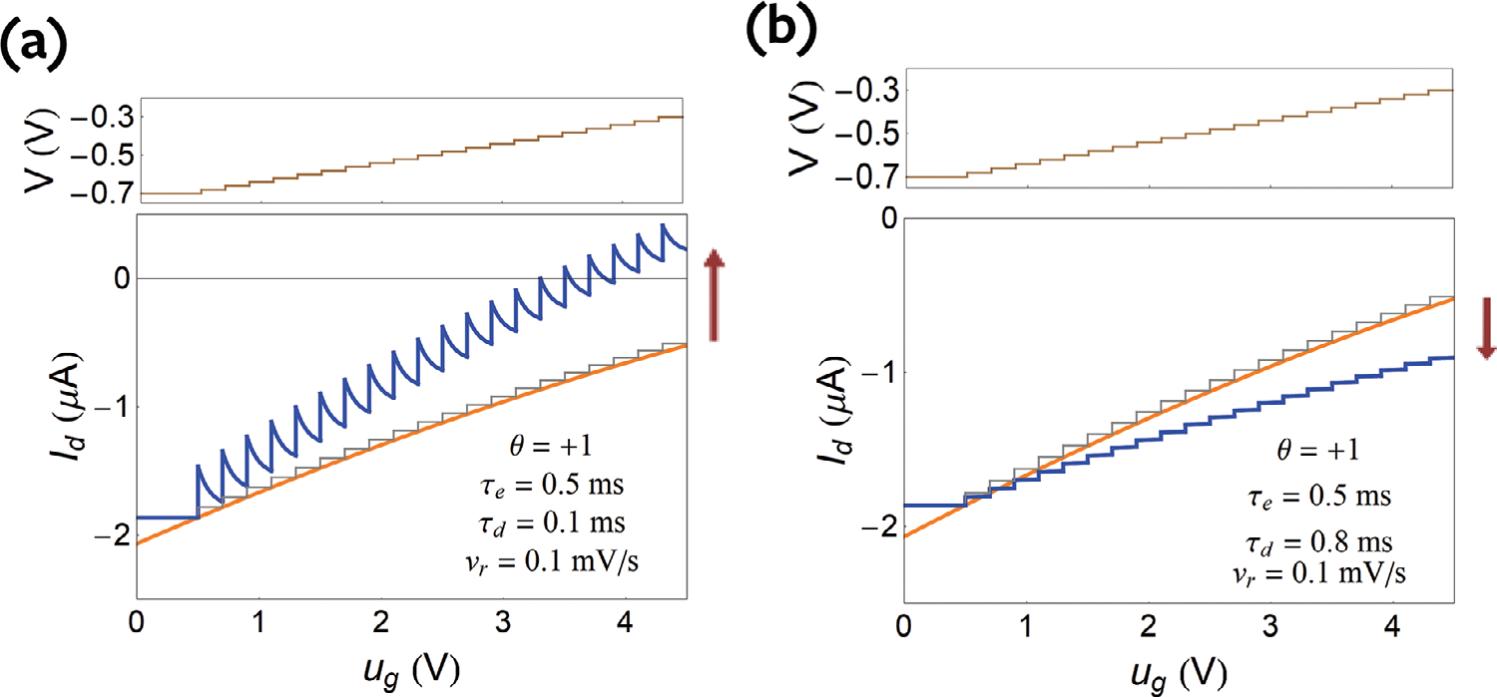
Undoped semiconductor for θ  =   +1. a–c) Increasing the drain current by small voltage steps Δ*V*  =  0.02 *V* starting at *V*_0_ =   −0.7 V (*t*_0_ =  0.5 s) with velocity *v_r_* at time steps Δ*t*  =  Δ*V*/*v_r_*, at increasingly faster rates from (a) to (b). The grey step line is an immediate current response, the orange line is the equilibrium current–voltage curve, and the red arrow in (c) is the final hysteresis current. The same parameters as Figure 2 and τ_*d*_, τ_*e*_ as indicated.

Hysteresis in current–voltage transfer curves are usually analyzed by applying the time‐dependent voltage with constant velocity *v_r_*,

(79) $$u_{g}=v_{r}\;t$$

for a go‐and‐return cycle in the voltage range of interest. However, in practice, the instrument develops small steps of voltage at time steps Δ*t*  =  Δ*V*/*v_r_*, as shown in **Figure** **6** for θ  =   −1 and in **Figure** **7** for θ  =   +1.

From the transient response in Figure 2 we can deduce the hysteresis effect that will happen due to ionic charging. As we have mentioned, the time of equilibration is τ_*d*_ in all cases. If the time step is long, the current reaches equilibrium at each time, and there is no hysteresis as in Figure 6a. Now if we apply a voltage step, but at time *t* ≈ τ_*d*_ or shorter apply a second step, the current in Figure 2b obtains an excess with respect to the equilibrium line. This is accumulated up to the red arrow as shown in Figure 6c. Hence in the forward direction, the measured current will be *larger* than the equilibrium current, and the hysteresis current will be larger when *higher* sweep velocity is applied.

In a previous review, two types of hysteresis have been distinguished.^[^ ^74^ ^]^ In capacitive hysteresis, the forward current is larger than the reverse current, as in Figure 6c, and in inductive hysteresis, the inverse situation is obtained. In two‐contact devices, these features are caused by the dominance of either capacitor or inductor impedance.

In the measurement of Figure 1b the situation is different, as the vertical and horizontal currents are measured in different contacts. Nevertheless, here we keep the denomination of “capacitive” or “inductive” hysteresis for practical discussion, but the underlying equivalent circuit has not been developed yet, and requires a separate analysis that will be presented elsewhere.

Let us formulate the model Equations (55) and (56) under the perturbation (82), as follows

(80) $$I_{d}=-\theta \frac{q\;L}{\tau_{e}}A-qLf\frac{dA}{dt}$$

(81) $$\tau_{d}v_{r}\;\frac{dA}{du_{g}}=A_{eq}\;\left(u_{g}\right)-A$$

The *I_d_* − *u_g_* curves are obtained by integration of Equation (81) to find *A*, then plotting the current in Equation (80). The hysteresis loops corresponding to Figure 6b,c, are shown in Figure 6d. The hysteresis effect becomes larger at increasing scan rate.

**Figure 8:**
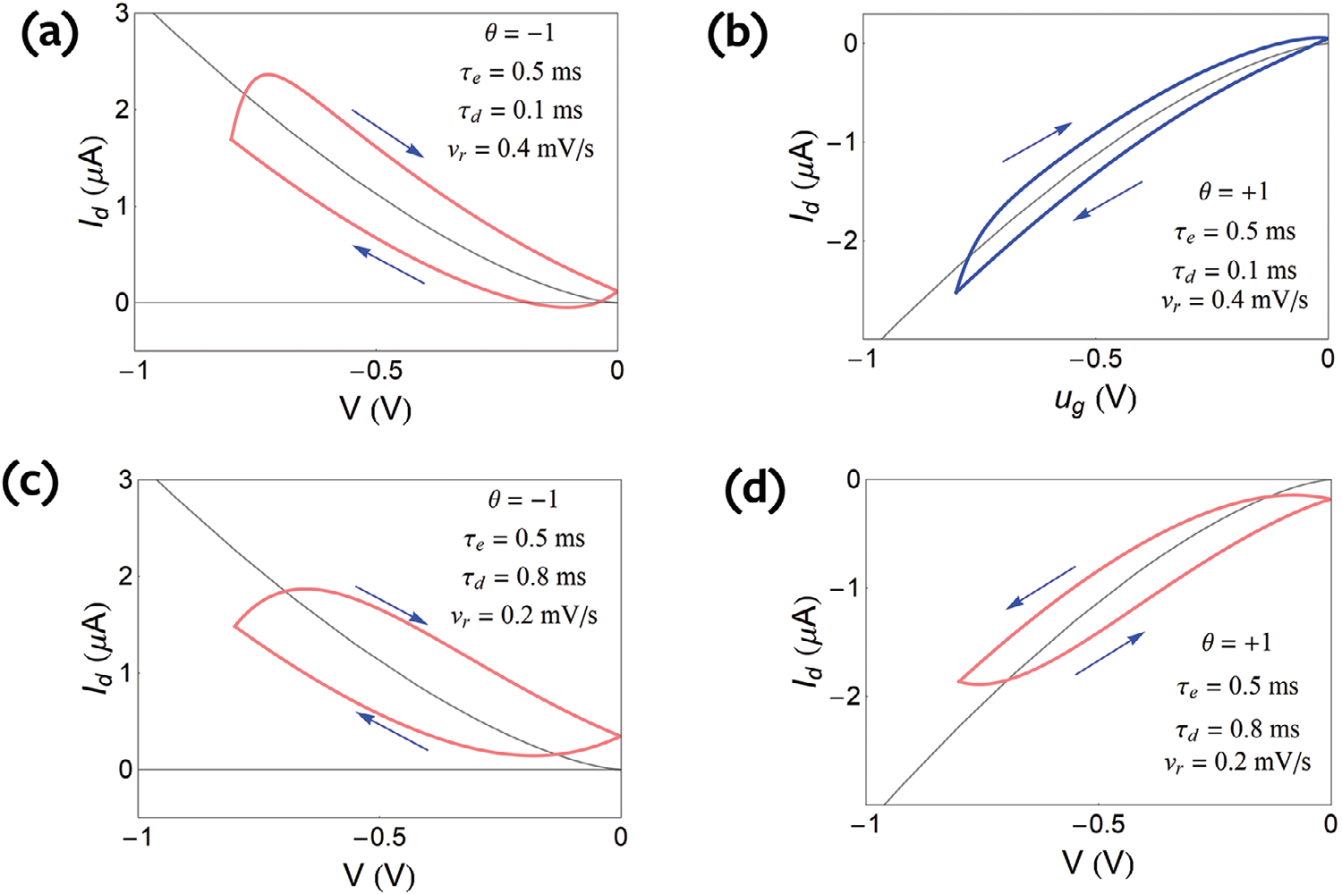
Undoped semiconductor. Continuous hysteresis loops at the indicated scan rates. The same parameters as Figure 2 and θ,  τ_*d*_, τ_*e*_ as indicated.

The four types of loops according to the four decay types of Figure 2 (depending on the sign of the *u_ds_* and the dominant relaxation time) are shown in **Figure** **8**. We note that in Figure 8a–c, (marked in pink line) the hysteresis is similar as it consists of an increase of the absolute value of the current in the forward direction, with respect to the equilibrium current, and a decrease in the reverse direction. This is the case of capacitive hysteresis in the general classification.^[^ ^32^, ^74^ ^]^ However Figure 7b and 8b show a different behavior (marked in blue line), in which the current at forward bias is less than the equilibrium current. This is the case of inductive hysteresis.^[^ ^32^, ^74^ ^]^

For the doped semiconductor, the hysteresis curves are obtained by solving the equations

(82) $$I_{h}\;\left(L\right)=-\theta \frac{qL}{\tau_{e}}\left(P_{0}-M\left(u_{g}\right)\right)+qfL\frac{dM}{dt}$$

(83) $$\tau_{d}v_{r}\;\frac{dM}{du_{g}}=M_{eq}\;\left(u_{g}\right)-M\left(u_{g}\right)$$

**Figure 9:**
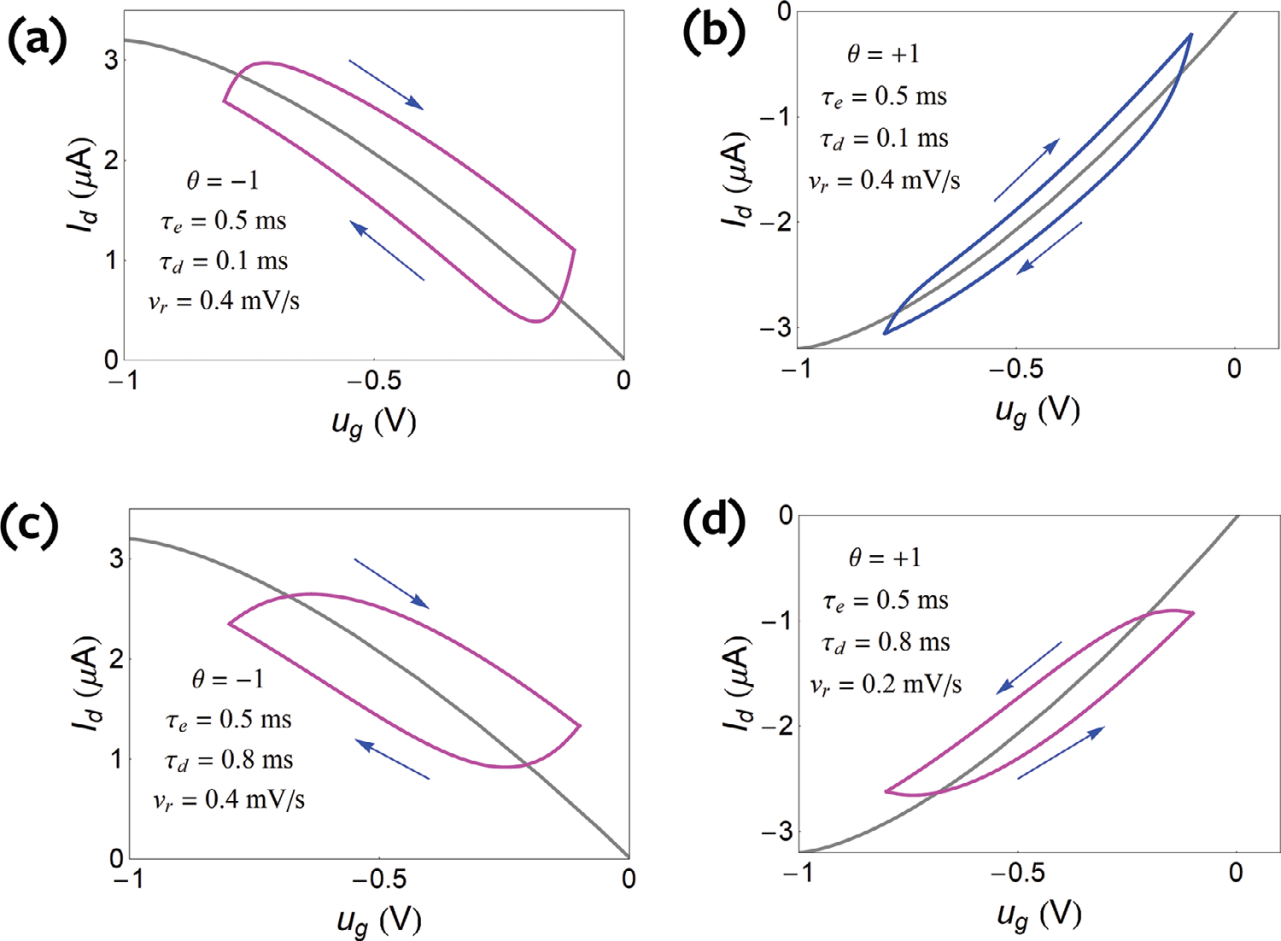
Doped semiconductor. Continuous hysteresis loops at the indicated scan rates. The same parameters as Figure 4 and θ,  τ_*d*_, τ_*e*_ as indicated.

The results corresponding to the step decays of Figure 4 are shown in **Figure** **9**. In correspondence with the undoped case, we obtain three cases of capacitive hysteresis, a, c, d (purple), and one case of inductive hysteresis, b, (blue).

## 5 Experimental Section

According to the electrochemical model Equations (80) and (81), four types of hysteresis loops in I_d_ versus u_g_ plots (transfer curves) were classified, which are determined by the sign of the voltage drop u_ds_ (θ) and the ratio of characteristic times τ_e_ and τ_d_. In order to experimentally validate the feasibility of hysteresis loops in real devices, the transfer curves of OECTs based on undoped semiconductors under various conditions were measured. For this purpose, OECTs with the channel based on a P3HT layer were fabricated and characterized.

P3HT, a well‐studied thiophene‐based conjugated polymer, has found applications in various electronic devices, including solar cells,^[^ ^75^, ^76^ ^]^ LEDs,^[^ ^77^ ^]^ and organic transistors.^[^ ^78^, ^79^, ^80^ ^]^ To construct the channel of an OECT, a layer of P3HT was cast onto a glass substrate with prepatterned ITO drain/source electrodes using a spin‐coating technique. The spin‐coated P3HT layer was intentionally not annealed to maintain its amorphous structure. According to research by Ginger and colleagues,^[^ ^78^, ^79^ ^]^ a transistor based on amorphous P3HT functions is an electrochemical transistor, whereas a highly crystalline P3HT bulk can impede ion intake, leading to the device operating as an electrolyte‐gated field‐effect transistor.

### 5.1 Methods

P3HT (LT‐S909) was purchased from Luminescence Technology Corp. Interdigitated prepatterned ITO glass substrates (S161: width × length: 30 mm × 50 µm) from Ossila served as the source‐drain electrodes.

The P3HT solution was prepared by dissolving 25 mg of polymer in 1 mL of chlorobenzene within a glovebox with an inert atmosphere. The solution was stirred for 3 h at 45 °C before spin‐coating. Prior to use, the ITO‐patterned source‐drain substrates underwent rigorous cleaning. Initially, the substrates were sonicated in deionized (DI) water with detergent for 10 min, followed by rinsing three times with DI water. Subsequently, they were sonicated in acetone and isopropyl alcohol (IPA) for 10 min each. Finally, the substrates were dried using nitrogen flow and treated with UV‐ozone for 15 min to remove any residual organics and enhance substrate surface wettability.

P3HT channel layers were deposited using a spin‐coating technique. A volume of 30 µL of the P3HT solution at 45 °C was dispensed onto the substrate spinning at a rate of 1000 rpm and kept rotating for 1 min. The as‐cast P3HT films were utilized without annealing, maintaining them in an amorphous state. Previous studies have shown that amorphous channel layers facilitate the penetration of anions under appropriate gate bias voltage, while organic transistors based on crystalline P3HT layers primarily function as electrolyte‐gated transistors.

A 10 m aqueous solution was employed as the electrolyte, with an Ag wire serving as the gate electrode. In addition, 0.1 M of potassium hexafluorophosphate (F_6_KP) in acetonitrile was also used as an electrolyte. IV curves were measured using a two‐channel Keithley source meter controlled by a customized LabVIEW program. Transfer curves of the devices were measured with gate voltages (u_g_) ranging from −0.1 to 0.5 V at various scan rates (V_r_) and drain biases (V_ds_). During the transfer curve measurements, the drain bias (V_ds_) was held between −0.2 and 0.2 V. This was done to minimize the influence of the drain (or source) potential and to ensure relatively uniform doping along the channel length.

### 5.2 Results

Transfer curves of P3HT OECTs were obtained by sweeping the gate bias (u_g_) from −0.5 to 0.1 V at various scan rates (v_r_). In the study, all parameters affecting τ_e_ and τ_d_ were fixed, except V_ds_. The electronic characteristic time is inversely proportional to V_ds_, and by decreasing V_ds_, the condition of τ_e_ > τ_d_ could be possibly reached. The transfer curves were probed at different drain biases to adjust the characteristic times τ_e_.

To ensure that the device mostly remains in the linear regime, transfer curves were measured at low V_ds_ (ranging from 25 to 200 mV). The output curve of P3HT OECT is presented in supplementary information (Figure S1, Supporting Information).

**Figure 10:**
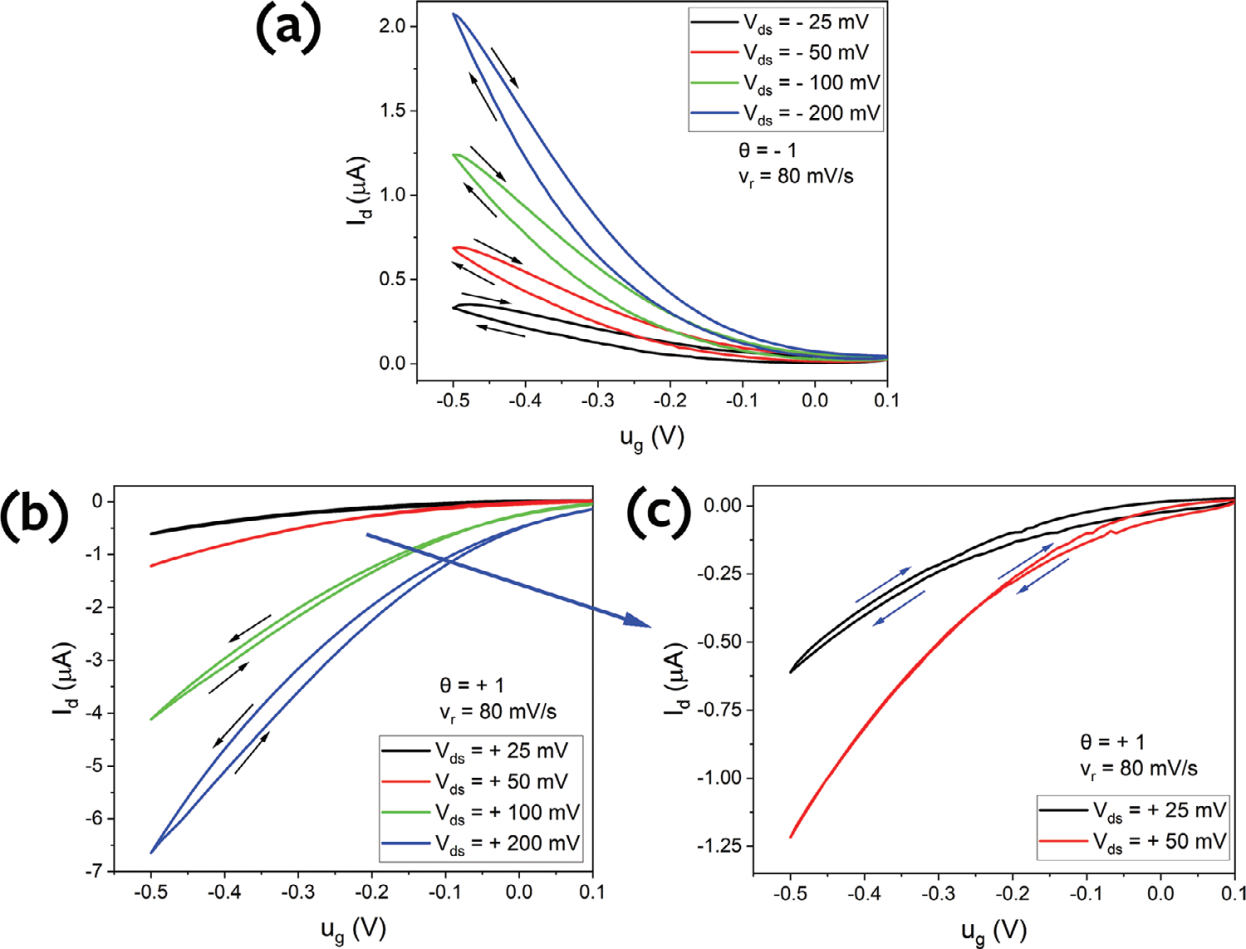
Transfer curves of P3HT OECT (the accumulation OECT with the initially undoped channel) at different values of drain‐source voltage. a) for θ = +1; b) for θ = −1; c) magnified (b) plots.

At first, P3HT OECT was considered with KCl aqueous solution. The transfer curves measured at various values and polarities of V_ds_ (at θ = −1 and θ = +1) are depicted in **Figure** **10**. As seen in Figure 10a, for θ = −1, the hysteresis loops exhibit a capacitive trend at all values of V_ds_ ranging from −25 to −200 mV, which is consistent with the theoretical prediction in Section 4. As demonstrated by plotting Equations (80) and (81) in Figure 8a, for both ratios of electronic and ionic characteristic times (τ_e_ > τ_d_ or vice versa), for θ = −1, hysteresis loops exhibit only capacitive behavior.

In the case of θ = +1, the hysteresis model predicts the possibility of observing an inductive hysteresis in the transfer curve of the OECT. According to Figure 8b, inductive hysteresis appears only for θ = +1 when the electronic relaxation time is dominant (τ_e_ > τ_d_). Experimentally, this condition can be achieved by increasing the channel length and decreasing V_ds_ (Equation 39), or by decreasing the channel thickness and increasing an anion diffusion coefficient (Equation 51).

Indeed, as shown in Figure 10b,c, for θ = +1, the decrease of V_ds_ switches the hysteresis type. At relatively high V_ds_, capacitive hysteresis was observed in the transfer curve, whereas at very low V_ds_ (at 25 mV), it becomes inductive in accordance with the analytical model. In fact, a shrinkage of capacitive hysteresis was observed with decreasing V_ds_. However, it should be noted that this phenomenon also could be explained by residual doping of P3HT, which will be discussed in detail in Section 6.

**Figure 11:**
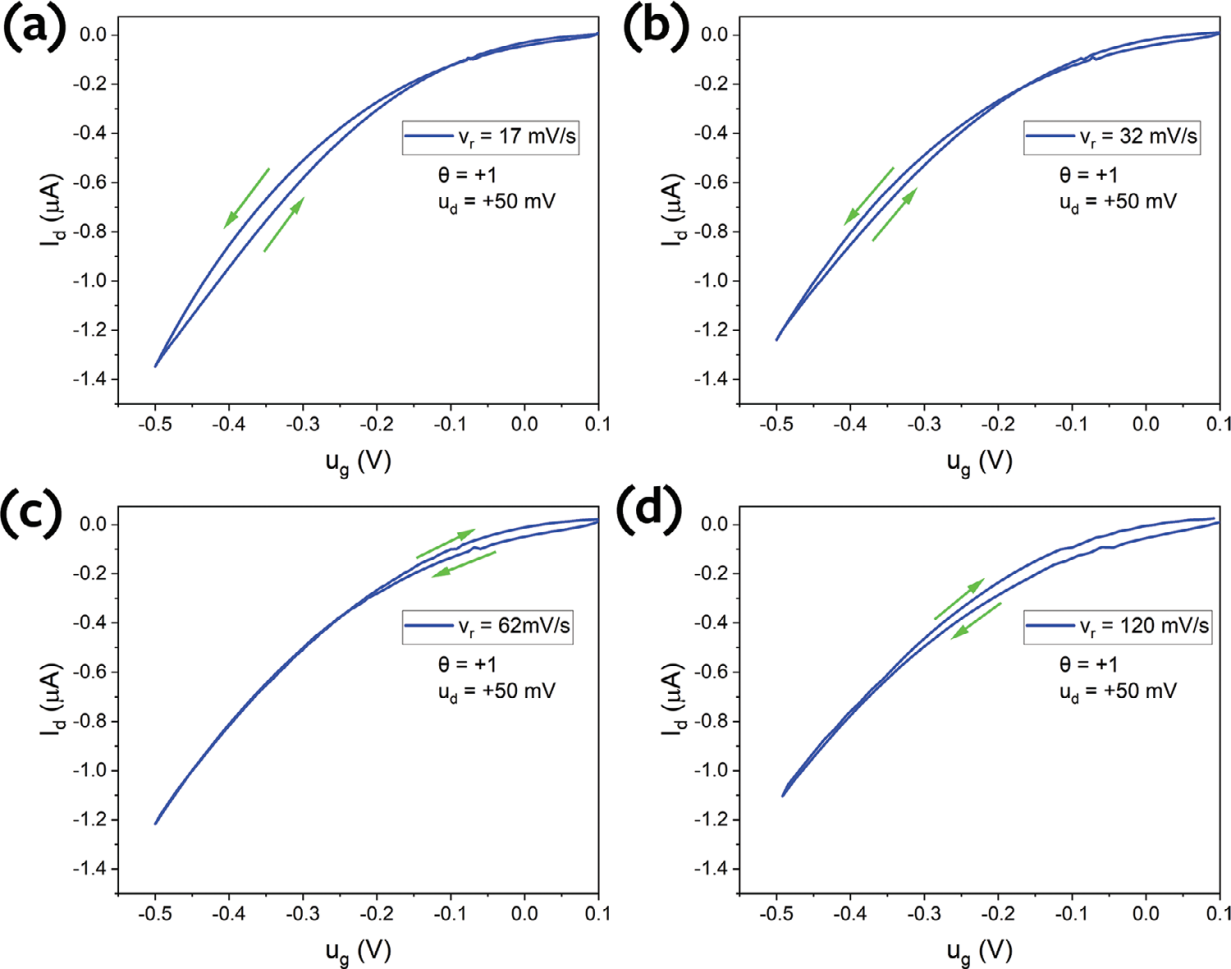
Transfer curves of P3HT OECT for θ = +1 and u_d_ = +50 mV at various scan rates.

In the case of θ = +1, as shown in Figure 10c, inductive hysteresis was detected at very low V_ds_. However, it also depends on the scan rate (v_r_). Inductive hysteresis is clearly observed at higher v_r_. As seen in **Figure** **11**, at relatively low v_r_ and low V_ds_, the hysteresis exhibits a capacitive pattern. At a fixed V_ds_, the capacitive hysteresis switches to inductive hysteresis at higher v_r_. When V_ds_ is maintained at 50 mV, the switch between types of hysteresis was observed at v_r_ = 62 mV s^−1^, and at v_r_ = 120 mV s^−1^, the hysteresis was predominantly inductive.

**Figure 12:**
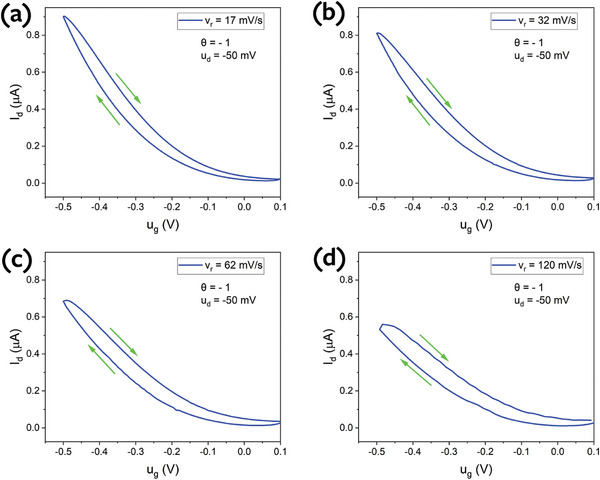
Transfer curves of P3HT OECT for θ = −1 and u_d_ = −50 mV at various scan rates.

For θ = −1, the increase in scan rate does not lead to a change in the hysteresis type (see **Figure** **12**). An increase was only observed in the hysteresis strength and a decrease in the overall current level by increasing v_r_, which was consistent with the theoretical model (Figure 6d).

In addition, the transfer curves of P3HT OECT were measured using an organic‐based electrolyte. The electrolyte used was potassium hexafluorophosphate (F_6_KP) in acetonitrile. Acetonitrile was chosen because it swells P3HT more effectively than water, facilitating the injection of anions. Additionally, hexafluorophosphate anions [PF₆]⁻ efficiently penetrate the P3HT bulk.^[^ ^81^ ^]^

As the case with KCl aqueous solution, the hysteresis type of the transfer curve in OECT with the F_6_KP acetonitrile electrolyte depends on the sign of θ and the value of the drain bias. At positive θ and relatively low V_ds_ of +50 mV, the hysteresis of the transfer curve was inductive (Figure S2a, Supporting Information). When V_ds_ increases, the hysteresis type switches to the capacitive pattern (Figure S2b, Supporting Information). Conversely, at negative θ and relatively low V_ds_ of −50 mV, the hysteresis was capacitive. Even at a more negative V_ds_ of −200 mV, the hysteresis remains predominantly capacitive.

### 5.3 Discussion of Experimental Results

The analysis of the transfer curves revealed hysteresis phenomena consistent with the model of the preceding sections. However, as already discussed, the model was based on the assumption of limitation by ion diffusion kinetics, while in general other possibilities exist, as commented in Figure 5b. To obtain the physical origin of the observed hysteresis effects, a more extensive investigation was needed, that addresses the transient currents in combination with hysteresis. This study was beyond the scope of the present paper. Such a more general study can also provide insight into the determination of the factor *f*. In Equation (70) *f* influences some terms of the transient current, thus a detailed kinetic analysis of the different characteristic times in the systemis needed.

## 6 Simulation of Transients and the Related Hysteresis

The purpose of this Section is to bridge between the electrochemical model and experimental data using a device simulation that can include many details of the device structure. We start with a simulation that aligns with the chemical physics picture used by the electrochemical model. In terms of the 2D device simulations,^[^ ^35^ ^]^ these translate to the following:

1. The electrolyte acts as an infinite supply of ions. We set its thickness to 200 µm.
2. The electrolyte presents no serial resistance. We set the ions’ diffusion coefficient in the solution at least six orders of magnitudes above that of the diffusion in the 100 nm thick semiconductor.
3. There is no voltage drop at the gate electrode interface. The gate is ohmic to anions, and the anions density at the gate interface is set to its value in the solution. The contact acts as a reflecting mirror (nonreacting) for the cations.
4. The ionic double‐layer capacitance (C_dl_) at the source and drain electrodes is negligible. We set the effective double‐layer thickness at 0.2 nm (i.e., areal capacitance is ≈10^−5^F cm^−2^), and the contact overlap with the semiconductor (L_c_) is kept well below 1 µm.
5. To avoid contact‐limited injection, the source and drain contact barriers are set low enough such that the hole's current is not reduced due to the short overlap (100 nm).^[^ ^82^ ^]^
6. When the ions enter the semiconductor, they do not create a space charge that would impede their diffusion into the film. To ensure a fast supply response of the compensating holes, we set their mobility at 5 cm^2^ V^−1^ s^−1^ (For L = 50 µm and V_ds_ = 0.1 V it results in t_e_ = 0.05 ms).
7. The mutual attraction, through the Poisson equation, does not affect the dynamics. The dynamic response is evaluated at low ion densities (<10^18^ cm^−3^) where the attraction is low.

**Figure 13:**
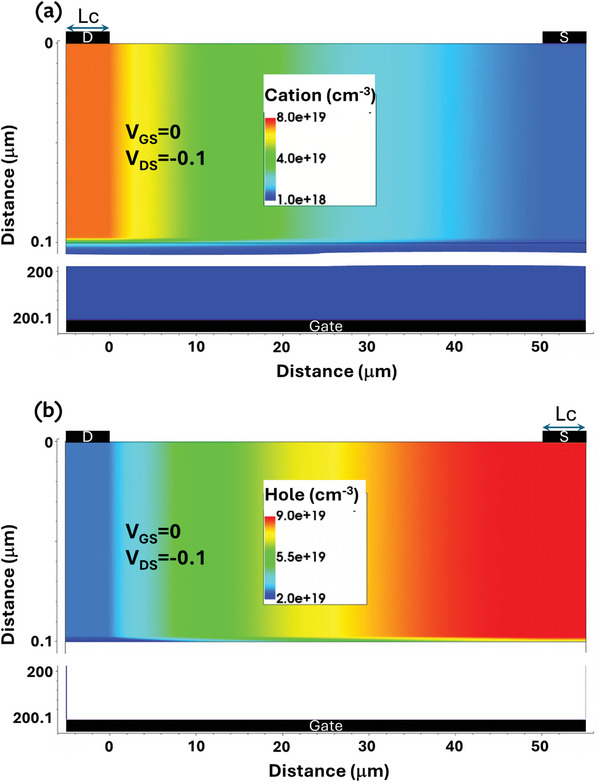
The simulated device structure containing the parameters of a P‐doped (10^20^cm^−^3) device. a) Cation density b) Hole density. V_gs_ = 0 V, V_ds_ = 0.1 V, W = L = 50 µm.

**Figure** **13** shows the simulated device structure (w = L = 50 µm), on top of which are results from a simulation of a p‐doped semiconductor (10^20^ cm^−3^) and an electrolyte ion concentration of 10^18^ cm^−3^ (≈2 millimolar). Note that the choice of coordinates in Figure 13 implies that for u_d_ < u_s_, the current is positive. Figure 13a,b show the cation a) and hole b) density distribution under bias conditions of V_gs_ = 0 and V_ds_ = 0.1 V. The hole and cation distribution shown in Figure 13 agree with previously reported simulation results. To follow the notation of the electrochemical model, the I_ds_ are positive here.

**Figure 14:**
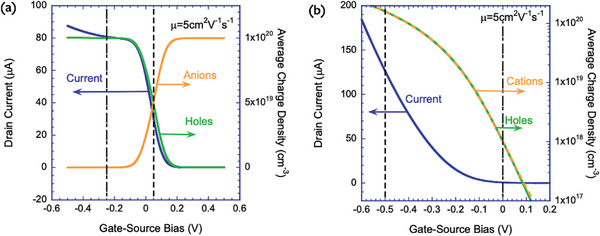
Current (blue), hole (green), and ion (orange) density as a function of the gate voltage. a) doped semiconductor. b) undoped semiconductor. The dashed‐dotted and the dashed black lines mark the voltage at which the low ion‐density and high ion‐density time‐dependent responses are evaluated.

**Figure** **14** shows the steady‐state current–voltage (blue line) response of devices based on doped a) and undoped b) semiconductors. The average density of the hole (green line) and ions (orange line) in the semiconductor are plotted on the right axis. For the P‐doped (10^20^ cm^−3^) device, we note that the hole density is a mirror image of the anion density following holes = doping–anions. For the undoped semiconductor (Figure 14b), the holes, cations, and the current have a perfectly overlapping shape. Hence, we took the opportunity and plotted the charge densities on a log scale.

In the following sections, we will present transient responses in two regimes. The first is the regime where the ion density within the semiconductor is low (<10^18^ cm^−3^), and we mark the relevant working points with dashed‐dotted lines in Figure 14. The second regime is for high ion density (>10^19^ cm^−3^) with the relevant working point marked by a dashed line. Figure 14b shows that the current rises for gate‐source bias below −0.2 V while the average hole density seems fixed. As reported in ref. [36] this is the signature of the thin hole channel generated by anions accumulation at the semiconductor interface.

### 6.1 Transient Response at Low Ion Density

**Figure 15:**
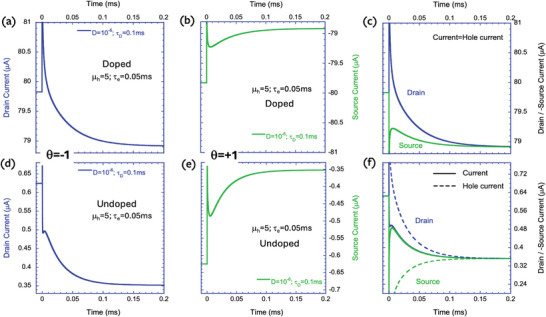
Transient response of the drain current (V_ds_ = 0.1 V) to a small step in the gate voltage after being stabilized at the bias marked with dashed‐dotted lines in Figure 14. a–c) P‐doped (10^20^ cm^−3^) semiconductor. d–f) Undoped semiconductor. a,d) drain current. b,e) source current. c,f) drain current and source current times −1.

To test for agreement between the semiconductor device simulations and the electrochemical model, we performed a transient response analysis at bias levels where the ion's density is low (dashed‐dotted lines in Figure 14). To ensure that the densities stay low throughout the response, we performed a small signal analysis using a +30 mV step voltage at t = 0. **Figure** **15** shows the transient response for devices based on P‐doped (top raw) and undoped (bottom raw) semiconductors. The left column shows the drain current (θ  =  1) and the middle one shows the source current (θ  =   − 1). We used hole mobility of µ_*h*_ =  5 *cm* ^2^ *V* ^−1^ *s* ^−1^ (τ_*e*_ =  0.05*ms*) and ion diffusion constant of D = 10^−6^ cm^2^ s^−1^ (τ_*d*_ =  0.1*ms*). Since τ_*e*_ < τ_*d*_ the simulations of the doped device, Figure 15a,b correspond to Figure 4f,g, respectively. We note that the change in the shape of the responses between Figure 15a,b agrees with the electrochemical model. Also, the stabilization time corresponds nicely to the ion's diffusion time (τ_*d*_). The difference between the source and drain currents during transients is best visualized in Figure 15c. The fact that the source drives less current than the one extracted at the drain is the signature of the semiconductor being discharged following the positive bias step. The point where the source and drain current converge marks the end of the transient.^[^ ^23^, ^64^ ^]^

Moving to the undoped device, Figure 15d,e correspond to Figure 2f,g, respectively. We find that Figure 15d does not agree with Figure 2f, and when we place the source and drain currents next to each other (Figure 15f), it seems as if no discharge takes place. This discrepancy suggests that the current of the undoped device is not just the hole current and that there is a significant contribution of displacement current (ε dE/dt). With the aid of the simulation, we isolated the hole currents and plotted them as dashed lines in Figure 15f. As they should, the hole currents are different enough to indicate a discharge following the step in the gate voltage. The electric field that is varying is the one between the source and drain even though V_ds_ is constant. The nonuniformity of the ion's influx creates variations in the electric field while keeping the integral (i.e., V_ds_) constant.

**Figure 16:**
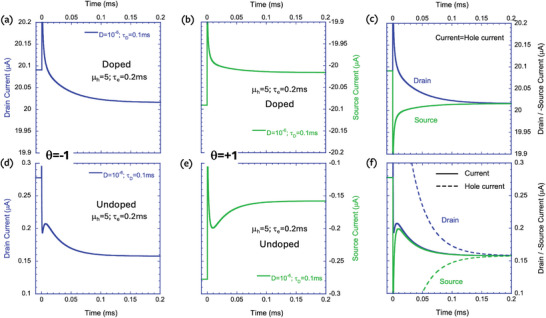
Transient response of the drain current (V_ds_ = 0.025 V) to a small step in the gate voltage after being stabilized at the bias marked with dashed‐dotted lines in Figure 14. a–c) P‐doped (10^20^ cm^−3^) semiconductor. d–f) Undoped semiconductor. a,d) drain current. b,e) source current. c,f) drain current and source current times −1.

Our next goal is to verify if making τ_e_ larger such that τ_*e*_ > τ_*d*_ inverts the slope of the source current (θ = +1), which would indicate that the hysteresis loop reversed direction. As with Figure 15, we find in **Figure** **16** that the doped device (top raw) agrees with the electrochemical model while the undoped one (bottom raw) does not. Note that in Figure 16b, the current flipped direction, and decreases toward the steady state value. The nonuniformity of the undoped device manifests itself with the source current (θ = +1, Figure 16e) still rising toward the steady state. This poses a potential problem since the experimental data for the undoped P3HT device shows inverted hysteresis at low V_ds_ bias. To address this issue, we simulate, in the following section, a device with a nonreacting gate electrode, as in the experiments.

### 6.2 Hysteresis Response and Comparison to Experiments

**Figure 17:**
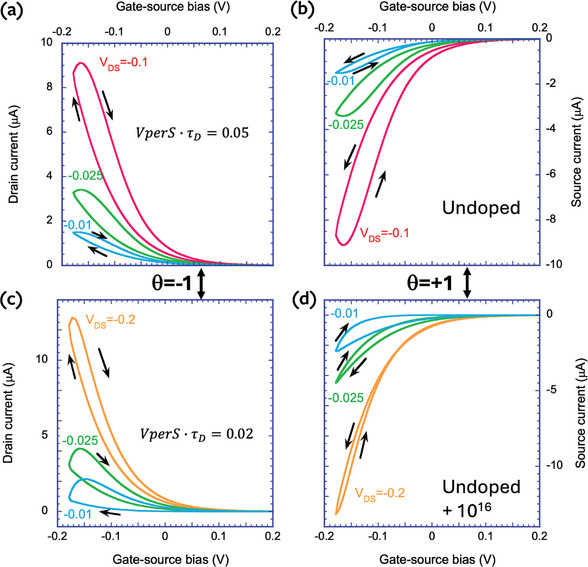
The drain (left) and source (right) currents' hysteresis response to a voltage sweep in the gate voltage after being stabilized at the initial bias (V_gs_ = 0.5 V) and for several drain‐source voltages (see Figure). a,b) The semiconductor is undoped, and the sweep speed is 0.05/t_d_. c,d) The semiconductor is undoped but with residual P doping of 10^16^ cm^−3^, and the sweep speed is 0.02/t_d_.

This subsection addresses the transient response issue of predicting that the hysteresis loop for θ = +1 (source current) should not change direction for the undoped device at low V_ds_ bias. This is important since the experimental data of the P3HT semiconductor (Figure 10) show the hysteresis changing direction and low V_ds_ bias (θ = +1, V_ds_ > 0). **Figure** **17**a,b show the corresponding simulated hysteresis loops at several V_ds_ values for the drain and source currents, respectively. We chose hole mobility (5 cm^2^ V^−1^ s^−1^) and ion diffusion (10^−6^ cm^2^ s^−1^) so that at V_ds_ = 50 mV, τ_*e*_ = τ_*d*_  =  0.1 *ms*. The scan rate was such that when multiplied by the ion diffusion time (t_d_) it equals 0.05 V. As expected from the simulated transient response, the hysteresis loop of the source current (θ = +1) does not change its direction at low drain‐source bias.

Examining the experimental data (Figure 10), we note that there is a sizable residual current at positive gate‐source bias. This is the signature of residual doping, which is common to P3HT films, with some of the reported values exceeding 10^16^ cm^−3^. To test if the residual doping could reduce the nonuniformity effects and recover the change in the hysteresis direction, we repeated the simulations with an added P‐doping of 10^16^ cm^−3^. The results are presented in Figure 17c,d for the drain and source currents, respectively. We note that, in agreement with the experimental data, the source (θ = +1) currents’ hysteresis loop direction changes at low V_ds_ values (Figure 17d). As stated in the experimental part, such a change in direction is not observed for the drain (θ = −1) current (Figure 17c).

### 6.3 Transient Response at High Ion Density

Having bridged between the electrochemical model and the experimental results, we use the 2D semiconductor device simulations to explore an effect that is not part of the Bernards–Malliaras model. At the very heart of any electrochemical model, one can find the charge neutrality concept. Since (space) charge accumulation creates a repulsive electric field for added charge, it is impossible to accumulate significant charge in a given volume. For the steady‐state solution, charge neutrality allows us to state that the density of holes equals (to an excellent approximation) the ion density. This simplifies the equations, as we only need to follow the ions.

However, such a conclusion is not necessarily correct for the transient. During the transient, the charge neutrality principle dictates that ions (holes) cannot penetrate the film unless holes (ions) arrive to compensate for the ions’ (holes’) charge. Namely, in the general case, the dynamics depend upon both t_e_ and t_d_. Naturally, the cross dependence, which is driven by the Poisson equation, is a function of the charge (ion) density. In the previous sections, we minimized this cross‐dependence by limiting the gate‐source bias to a range where the ion density is below 10^18^ cm^−3^. In the following figure, we will show results for the range in which the ion density is above 10^19^ cm^−3^.

**Figure 18:**
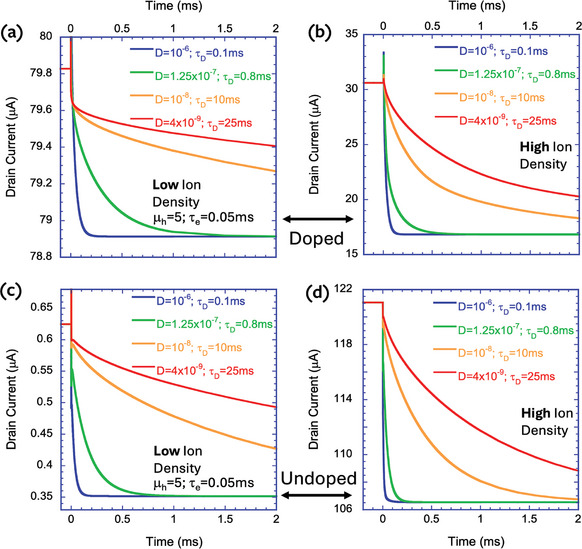
The transient response of the drain current to a small step in the gate voltage after being stabilized at the initial bias of V_ds_ = 0.1 V and V_gs_ was set according to Figure 14. a,b) Doped semiconductor. c,d) undoped semiconductor. a,c) Low ion density (V_gs_ was stabilized at the bias marked with dashed‐dotted lines in Figure 14). b,d) High ion density (V_GS_ was stabilized at the bias marked with dashed lines in Figure 14). For the low ion density, the stabilization time is similar to the ion diffusion time (t_d_) and for the high ion density, it is much faster.

In **Figure** **18** we look at the effect of the hole transit time being much shorter than the ion's diffusion time (τ_*e*_ ≪ τ_*d*_). We examine only the drain current (θ = −1), with the left column for the low‐density case and the right one for the high‐density. As noted in Figure 14a, for the doped semiconductor, the gate bias that results in low cation density is negative, and the one resulting in high cation density is positive. For the undoped case, the high anion density corresponds to more negative gate bias (Figure 14b). For comparison, we added results from longer diffusion times. Both Figure 18a (doped) and Figure 18c (undoped) show that the stabilization time constant is close to the ion diffusion time and the use of *exp*(− *t*/τ_*d*_), is justified. The high‐density results, which appear in the right column, are very different. The stabilization time constant still depends on the diffusion time, but it is now much shorter. Notably, the orange line representing the diffusion time of 10 ms exhibits a stabilization time of ≈2 ms. Apparently, the fast holes expedite the transport of the ions.^[^ ^18^ ^]^

**Figure 19:**
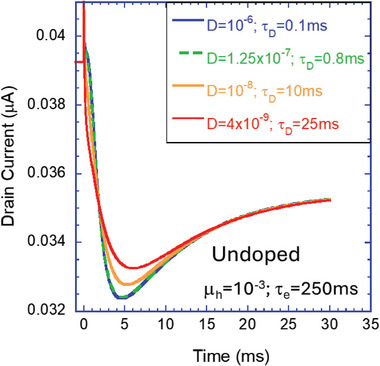
The drain current's transient response to a small step in the gate voltage after being stabilized at the initial bias (V_ds_ = 0.1 V). The hole transit time is much longer than the ion diffusion time (m_h_ = 10^−3^cm^2^ V^−1^ s^−1^, t_d_ = 250 ms). The semiconductor is undoped, and the response is at high ion density (dashed line in Figure 13).

While Figure 18 showed that holes entering fast would expedite the ions, we use **Figure** **19** to demonstrate that slow holes delay the ions’ response. In Figure 19 the hole mobility is reduced to 10^−3^ cm^2^ V^−1^ s^−1^ resulting in τ_e_ = 250 ms. All the lines, starting from τ_d_ = 0.1 ms (blue line) and moving all the way to τ_d_ = 25 ms (red line), converge together toward the steady state at longer than 30 ms. Again, it shows that at high ion density, the response time depends on both the hole transit time (τ_e_) and the ion's diffusion time (τ_d_).

## 7 Conclusion

We developed a general formulation for a 2D transmission line description of an OECT. The general model considers the horizontal transport of electronic carriers and vertical diffusion of compensating ions, without space charge effects. Then we provided an analytical time‐dependent model under assumptions of homogeneous charge distribution. This model is equivalent to Bernards–Malliaras conservation equation, extended by including the diffusion effect so that two different time constants have been identified, the horizontal hole transport τ_*e*_ and the vertical ion diffusion τ_*d*_, depending on transport coefficients, morphology, and horizontal field.

The model enables a basic classification of the transient response to a step voltage in terms of the sign of the current and the sign of the step. Based on these elementary responses we found the possible types of hysteresis behavior in transfer curves. The experimental results validate the theoretical model, demonstrating the influence of various parameters such as drain bias, scan rate, and polarity on the hysteresis behavior observed in OECT transfer curves. The analysis of realistic simulation results shows very good agreement with the simple model, hence the model produces a robust elementary classification of the dynamic responses of OECT that are mainly governed by ion diffusion and hole transport issues. However, additional effects occur in realistic simulation, since the carrier density affects the effective transport parameters and changes the results expected in single carrier parameter pictures.

These findings contribute to a deeper understanding of the underlying mechanisms governing the operation of organic electrochemical transistors.

## Conflict of Interest

The authors declare no conflict of interest

## Supporting information

Supporting Information

## Data Availability

The data that support the findings of this study are openly available in Zenodo at [DOI], reference number 10925192.
