# Supplementary material for: Switching Response in Organic Electrochemical Transistors by Ionic Diffusion and Electronic Transport
Source: Adv Sci (Weinh). 2024 Jul 25;11(36):2404182. doi: 10.1002/advs.202404182 (PMC11423187; doi:10.1002/advs.202404182)
Supplement: Supplementary file 1 — Supporting Information [file ADVS-11-2404182-s001.docx]

Supporting Information

Title: **Engineering 3D Scaffold-Free Nanoparticle-Laden Stem Cell Constructs for Piezoelectric Enhancement of Human Neural Tissue Formation and Function**

Author(s), and Corresponding Author(s)*: *Emma Claire James, Eva Tomaskovic-Crook^*^, Jeremy Micah Crook^3*^*

**Video S1.** Human neural spheroids loaded with barium titanate nanoparticles (BTNPs) for ultrasound-mediated piezoelectric stimulation. Confocal fluorescence microscopy z-projection of Poly-L-Lysine (PLL) coated BTNPs relative to plasma membranes of human neural stem cells (PLL-BTNPs: green, plasma membranes: red). PLL was labelled with FITC, and cell membranes (including intracellular membranes, liposomes, and lipoproteins) were labelled with VybrantTM CM-DiI cell labelling solution. Scale bar: 20 μm.

**Videos S2 and S3.** Uptake of barium titanate nanoparticles (BTNPs) by human neural spheroids for ultrasound-mediated piezoelectric stimulation. Time lapse imaging by confocal microscopy of BTNP uptake (BTNPs: green; cell membranes: red). BTNPs were coated with Poly-L-Lysine (PLL)-FITC, and cell membranes (including intracellular membranes, liposomes, and lipoproteins) were labelled with VybrantTM CM-DiI. Video S2 Scale bar: 50 μm.
